# Regulation of *RNA-Dependent RNA Polymerase 1* and *Isochorismate Synthase* Gene Expression in *Arabidopsis*

**DOI:** 10.1371/journal.pone.0066530

**Published:** 2013-06-17

**Authors:** Lydia J. R. Hunter, Jack H. Westwood, Geraldine Heath, Keith Macaulay, Alison G. Smith, Stuart A. MacFarlane, Peter Palukaitis, John P. Carr

**Affiliations:** 1 Department of Plant Sciences, University of Cambridge, Cambridge, United Kingdom; 2 The James Hutton Institute, Invergowrie, Dundee, United Kingdom; 3 Division of Environmental and Life Sciences, Seoul Women’s University, Seoul, Republic of Korea; National Taiwan University, Taiwan

## Abstract

**Background:**

RNA-dependent RNA polymerases (RDRs) function in anti-viral silencing in *Arabidopsis thaliana* and other plants. Salicylic acid (SA), an important defensive signal, increases *RDR1* gene expression, suggesting that RDR1 contributes to SA-induced virus resistance. In *Nicotiana attenuata RDR1* also regulates plant-insect interactions and is induced by another important signal, jasmonic acid (JA). Despite its importance in defense *RDR1* regulation has not been investigated in detail.

**Methodology/Principal Findings:**

In Arabidopsis, SA-induced *RDR1* expression was dependent on ‘NON-EXPRESSER OF PATHOGENESIS-RELATED GENES 1’, indicating regulation involves the same mechanism controlling many other SA- defense-related genes, including *pathogenesis-related 1* (*PR1*). Isochorismate synthase 1 (ICS1) is required for SA biosynthesis. In defensive signal transduction *RDR1* lies downstream of *ICS1*. However, supplying exogenous SA to *ics1*-mutant plants did not induce *RDR1* or *PR1* expression to the same extent as seen in wild type plants. Analysing *ICS1* gene expression using transgenic plants expressing ICS1 promoter:reporter gene (β-glucuronidase) constructs and by measuring steady-state *ICS1* transcript levels showed that SA positively regulates *ICS1*. In contrast, *ICS2*, which is expressed at lower levels than *ICS1*, is unaffected by SA. The wound-response hormone JA affects expression of Arabidopsis RDR1 but jasmonate-induced expression is independent of CORONATINE-INSENSITIVE 1, which conditions expression of many other JA-responsive genes. Transiently increased *RDR1* expression following tobacco mosaic virus inoculation was due to wounding and was not a direct effect of infection. *RDR1* gene expression was induced by ethylene and by abscisic acid (an important regulator of drought resistance). However, *rdr1*-mutant plants showed normal responses to drought.

**Conclusions/Significance:**

*RDR1* is regulated by a much broader range of phytohormones than previously thought, indicating that it plays roles beyond those already suggested in virus resistance and plant-insect interactions. SA positively regulates *ICS1*.

## Introduction

RNA silencing refers to a set of gene regulation mechanisms occurring in most eukaryotes, whereby transcript stability or translatability is suppressed in a sequence-specific manner, guided by small 19–24 nt RNA molecules [1], [2]. RNA silencing is an important component of anti-viral defense in plants [3], [4]. Double-stranded structures within viral RNA can be cleaved by dicer-like (DCL) nucleases to generate double-stranded small interfering (si)RNAs. In *Arabidopsis thaliana*, there are four DCL enzymes, of which DCL4 and DCL2 are the most important in the generation of virus-derived siRNAs [5], [6], [7], [8]. After further processing, single-stranded forms of virus-derived siRNA molecules associate with Argonaute (AGO) nucleases and direct AGO-catalysed slicing of complementary viral RNA molecules [9]. Of the ten AGOs encoded by the *Arabidopsis* genome, AGO1 is the primary ‘antiviral’ AGO, with secondary roles for AGO2, and in certain instances for AGO7 [10], [11], [12].

Another important feature of the anti-viral RNA silencing pathway in plants is referred to as amplification, whereby more virus-specific dsRNA substrates for DCLs are generated *de novo* by cellular RNA-dependent RNA polymerases (RDRs) [13]. The *Arabidopsis thaliana* genome encodes six RDRs, characterized by the DFDGD catalytic domain, of which RDRs 1, 2 and 6 are known to be involved in biogenesis of siRNAs [14]. In *Arabidopsis* and other plants, RDRs 1 and 6 contribute to antiviral RNA silencing, whilst RDR2 is involved in establishment of transcriptional gene silencing [8], [13], [15], [16], [17], [18], [19], [20], [21], [22], [23], [24]. RDRs also contribute to silencing mediated turnover of transcripts encoded by endogenous plant genes and transgenes [1], [25].

Xie and colleagues [15] reported that in tobacco (*Nicotiana tabacum*) *RDR1* gene expression is induced by the defensive phytohormone salicylic acid (SA). This was a notable finding because it provided for the first time a possible connection between RNA silencing and two well-studied resistance phenomena that are dependent upon SA-mediated signal transduction: (i) the hypersensitive response, a genetically defined and highly pathogen-specific defense; and (ii) systemic acquired resistance (SAR) a broad-spectrum resistance to pathogens that is often triggered by a hypersensitive response [26].

However, it was also reported by Xie *et al.* [15] that although knockdown of *NtRDR1* expression in transgenic tobacco enhanced the susceptibility of these plants to infection by tobacco mosaic virus (TMV) and potato virus X, resistance to these viruses could still be induced by treatment of the plants with exogenous SA. Subsequently, it was shown that *Arabidopsis* mutants compromised in *AtRDR1* expression showed normal responses to bacterial infection and normal SA-induced expression of *pathogenesis-related protein 1* (*PR1*: a marker for SA-induced resistance to bacteria, oomycetes and fungi), while no effect on SA-induced virus resistance in these *Atrdr1* mutants was reported [16]. Constitutive expression of the *Medicago truncatula RDR1* in *N. benthamiana* (a natural *rdr1* mutant: [18]) did not enhance SA-induced resistance or rescue chemically-induced resistance in plants compromised in induced resistance by expression of a mutant form of alternative oxidase [27]. Curiously, expression of *NtRDR1* in transgenic *N. benthamiana* plants enhanced, rather than ameliorated, infection by plum pox virus [28]. Thus, although it is possible that RDR1 may contribute to SA-induced resistance to certain viruses, it is not an indispensible component of anti-viral resistance, and in some cases its expression may enhance susceptibility.

The regulation of *RDR1* gene expression is not well understood. It was reported that TMV infection triggered increased *NtRDR1* transcript accumulation in the tobacco cultivar Xanthi (*nn* genotype) [15] but this could not be due to increased levels of SA, since infection of this cultivar with TMV does not induce SA accumulation [29]. Interestingly, in *N. attenuata*, *RDR1* was induced by jasmonic acid (JA) [30], a phytohormone that is often assumed to be antagonistic to SA-mediated defensive signaling [31]. JA regulates induced resistance to herbivorous insects, and experiments with transgenic *N. attenuata* plants deficient in *RDR1* expression showed that NaRDR1 regulates inducible genes conferring resistance to insect herbivory [32]. Diminishing *NtRDR1* expression in transgenic tobacco decreased the expression of several endogenous transcripts related to virus resistance including *Alternative Oxidase 1a* and *NtRDR6* [33]. Thus, in addition to its hypothesized role in enhancing antiviral RNA silencing [15], [16], RDR1 may play indirect roles in plant defense via silencing-mediated regulation of cellular mRNAs encoding resistance factors. In this study we have investigated in more detail the regulation of *AtRDR1* gene expression by SA and JA, and found that other phytohormones, including abscisic acid (ABA) and ethylene, trigger its expression.

## Results

### SA-induced *AtRDR1* Expression is NPR1-dependent

**Figure 1:**
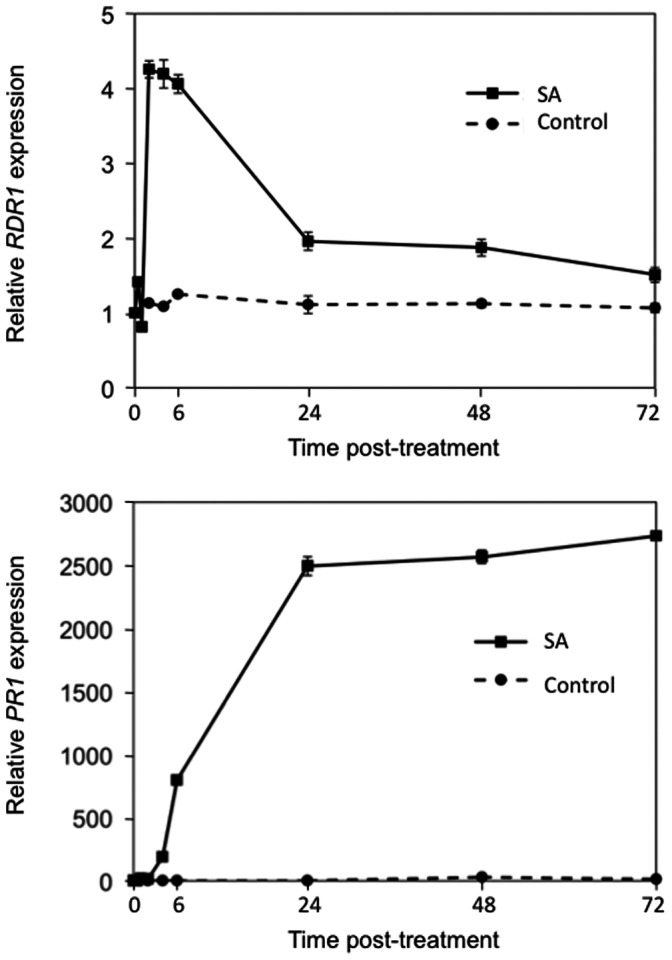
SA treatment causes transient induction of *AtRDR1* expression. *AtRDR1* expression and *AtPR1* expression in control and SA-treated *Arabidopsis* (Col-0) plants sampled at immediately before treatment (‘0’ time) and over a time course of 72 h. Error bars represent standard error of the mean.

Wild-type *Arabidopsis* plants (ecotype Col-0) were treated with 1 mM SA, then samples were taken over a time course spanning 72 hours (h) and were analysed by reverse transcription coupled with quantitative PCR (RTqPCR) for *RDR1* and *PR1* expression (Figure 1). In agreement with previous studies using northern blotting [16], *AtRDR1* transcript accumulation increased following SA treatment. In this study SA treatment peaked (at approximately four-fold basal level) between 2 and 6 h post-treatment before decreasing to approximately two-fold at 24 h post-treatment and returning to near starting levels by 72 h post-treatment (Figure 1A). The increase in accumulation of *AtPR1* transcripts confirmed that the treatment with SA had been effective (Figure 1). This is in contrast to the work of Yu and colleagues, who reported that *AtRDR1* induction took longer to become detectable (4 to 8 h) with no diminution of *AtRDR1* expression apparent at 24 h post-treatment, the point at which the analysis was terminated [16]. The current work shows that, in contrast to SA-induced *AtPR1* gene expression, the effect of SA on *AtRDR1* gene expression is transient (Figure 1).

**Figure 2:**
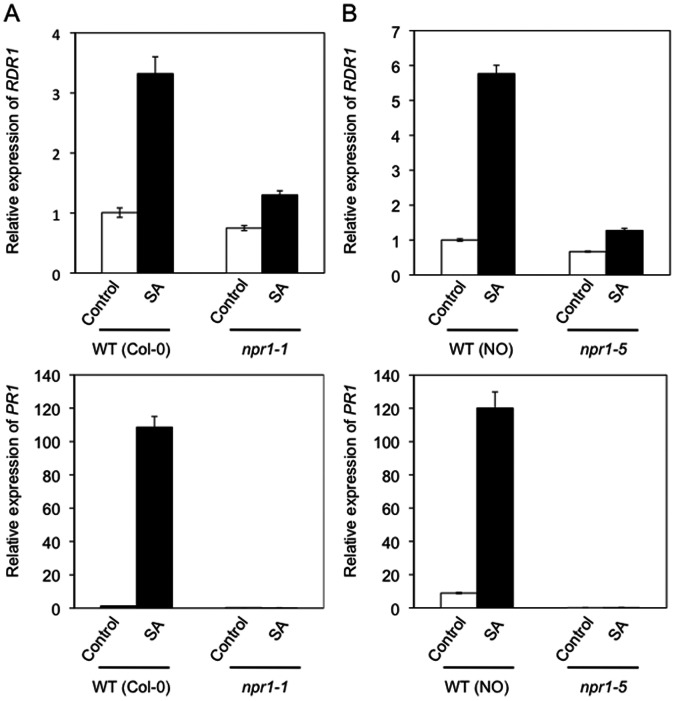
SA induces *AtRDR1* expression in an NPR1-dependent manner. (A) *AtRDR1* and *AtPR1* expression in ecotype Col-0 wild type and *npr1-1* control and SA treated plants 6 h after treatment. (B) *AtRDR1* and *AtPR1* expression in Nössen (NO) wild-type and *npr1-5* control and SA treated plants 6 h after treatment. Error bars represent standard error of the mean.

The transcriptional activator ‘Non-Expressor of PR proteins 1’ (NPR1) is required for *PR* gene induction and SAR against a wide range of microbial pathogens [34], although it is not required for SA-induced resistance to viruses [35], [36]. To determine if *RDR1* gene expression is NPR1-dependent, we used two independent mutant lines: *npr1-1* (Col-0 background: [37]) and *npr1-5*, which was originally named *salicylic acid insensitive 1* (*sai1*) (Nössen background: [38]), and examined *AtRDR1* transcript accumulation in plants at 6 h post-treatment with SA. As a control, the induction of *AtPR1* by SA, which is dependent upon NPR1, was also examined in wild-type and *npr1*-mutant plants. SA-induced *AtRDR1* expression in both *npr1* mutant lines was markedly lower than that in wild-type plants (Figure 2), indicating that it is NPR1-dependent. It was noted that in all experiments *AtRDR1* expression was consistently higher at 6 h post-treatment in plants of the Nössen ecotype than in plants of the Col-0 ecotype (Figure 2).

### *ICS1* Expression is Auto-regulated by SA and is Required for Maximal SA-Induced Expression of *RDR1*

**Figure 3:**
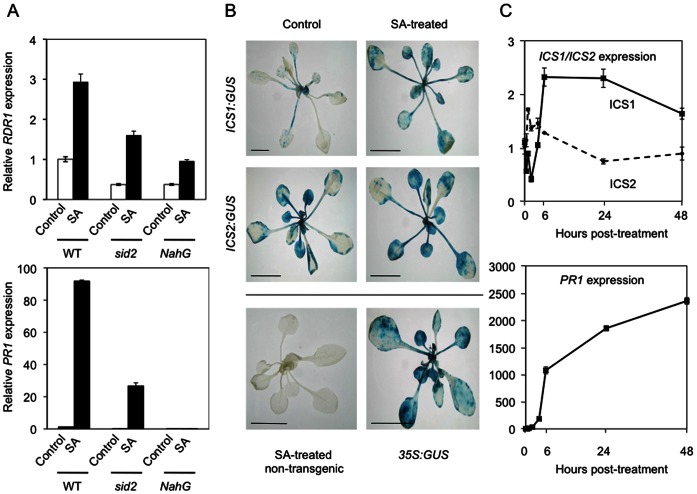
*AtICS1* expression is positively regulated by SA and is required for optimal expression of *AtRDR1* and *PR1* in response to exogenous SA treatment. (A) RTqPCR analysis of transcript accumulation for *AtRDR1* and *AtPR1* expression in *Arabidopsis* ecotype Col-0 wild type (WT), *sid2*-mutant (compromised in expression of *ICS1*) and *NahG*-transgenic plants 6 h post SA treatment. (B) Transgenic *Arabidopsis* plants harboring the promoter: reporter constructs *ICS1:GUS* and *ICS2:GUS* stained for GUS activity 24 h after control (water) or SA treatment. SA treated Col-0 WT and *35S:GUS* plants, 24 h after treatment are included as controls. (C) RTqPCR analysis of *AtICS1*, *AtICS2*, and *AtPR1* transcript accumulation in wild-type plants treated with SA, over a 48 h time course. Error bars represent standard error of the mean.

Plants of the *sid2* line are impaired in their ability to synthesize SA due to a lesion in the gene encoding the isochorismate synthase isozyme, ICS1, upon which *Arabidopsis* is dependent for the bulk of its stress-induced SA biosynthesis, and which is a key factor in the induction of SAR in this species [39], [40], [41]. It was observed during initial experiments on the role of NPR1 in *AtRDR1* induction that SA treatment of *sid2* mutant plants caused induction of less *AtRDR1* and *PR1* expression than was seen in wild-type plants (data not shown and Figure 3A). This result was unexpected since although these plants are compromised in their ability to produce SA, it was anticipated that addition of exogenous SA would rescue expression of the two SA-inducible transcripts. As expected, in plants of the transgenic *NahG* line, which expresses a bacterial salicylate hydroxylase [42], SA-induced accumulation of both transcripts was greatly diminished (Figure 3A).

We investigated the effect of exogenous SA application on expression of the *AtICS1* gene, as well as the other *Arabidopsis ICS* ortholog, *ICS2*. Transgenic plants harboring *ICS1:β-glucuronidase* (*GUS*) and *ICS2:GUS* promoter:reporter gene fusion constructs were treated with SA. *ICS1:GUS-*transgenic plants consistently exhibited increased GUS activity 24 and 48 h after SA treatment (histochemical analysis of 24 h samples are shown in Figure 3B). In contrast, GUS activity was already detectable in untreated *ICS2:GUS*–transgenic plants, and showed no induction at either time-point.

The responsiveness of *ICS* gene expression was investigated further by examining transcript accumulation for *ICS1* and *ICS2* using RTqPCR (Figure 3C). *ICS2* transcript accumulation increased transiently after SA treatment but decreased again by 6 h post-treatment. *ICS1* transcript accumulation increased by 2–2.5 fold within 6 h of SA treatment but this elevated level was sustained over 24 hours, followed by a gradual decline (Figure 3C). These results were consistent over three biological replicates. Therefore, *ICS1* gene expression appears to be positively auto-regulated by SA. ICS1 is the isozyme responsible for the bulk of SA biosynthesis [40], [43], [44] and unlike the gene for ICS2, the *ICS1* gene is stimulated in a sustained fashion by SA (Figure 3C). This positive auto-regulation of ICS1 expression by SA appears to explain why in *sid2* mutant plants the increase in *PR1* and *RDR1* gene expression triggered by exogenous SA was weaker than in wild-type plants (Figure 3A).

### Mock Inoculation Induced *RDR1* Expression

**Figure 4:**
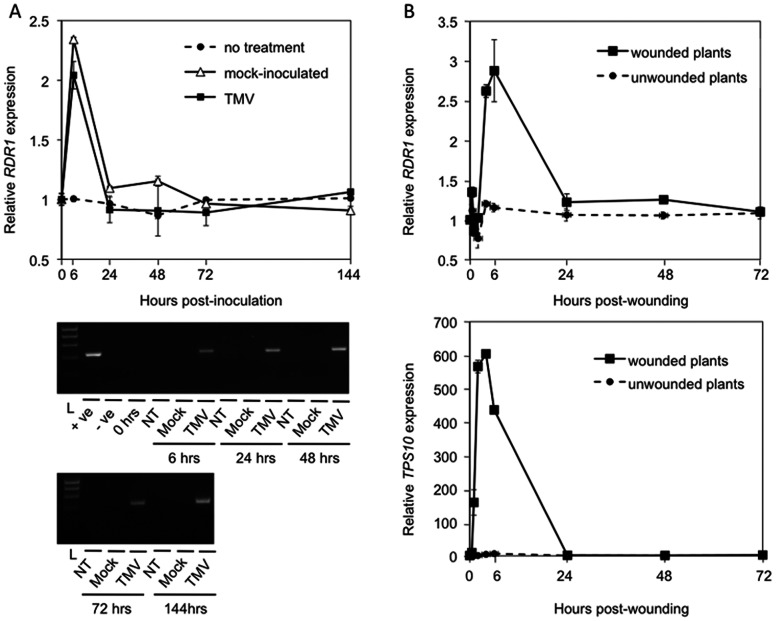
Wounding and mock-inoculation induces *AtRDR1* expression. (A) *AtRDR1* expression in TMV-infected, mock-inoculated (Mock) and untreated control (no treatment) *Arabidopsis* (Col-0) plants over the course of 144 h monitored by RTqPCR (Upper panel). Lower panel shows confirmation by RT-PCR of infection in TMV-inoculated compared with mock-inoculated and untreated (NT, no treatment). (B) RTqPCR of expression of *AtRDR1* and the wounding- and JA-responsive gene *AtTPS10* over 72 h following wounding. Error bars represent standard error of the mean.

TMV infection was reported to increase accumulation of *RDR1* transcripts in the inoculated leaves of *Arabidopsis* [16]. However, we found that the kinetics of *RDR1* transcript accumulation were similar in mock-inoculated and TMV-inoculated leaves (Figure 4A). In both cases, increased *RDR1* expression was transient, peaking and declining during the first 24 h following treatments and in the TMV-inoculated leaves there was no obvious relationship between *RDR1* expression and the kinetics of viral RNA accumulation (Figure 4A). This suggests that the process of inoculation, involving abrasion of the adaxial surfaces of the leaves with Carborundum, rather than virus infection *per se*, was responsible for increased *AtRDR1* expression.

To explore the possibility that *RDR1* expression was triggered by abrasion, further experiments were carried out to follow the expression of both *RDR1* and a well-characterized wound-induced gene, *terpene synthase 10 (TPS10)*, over a period of 72 h following mock inoculation (Figure 4B). Although the response of *RDR1* to mock-inoculation in terms of fold-increase in expression was at least two orders of magnitude less than the response of *TPS10*, the timing of expression following mock-inoculation was similar for both transcripts, supporting the idea that wounding had triggered increased *RDR1* gene expression in TMV-inoculated plants (Figure 4A,B).

### JA Induces *RDR1* Expression in a COI1-independent Manner

**Figure 5:**
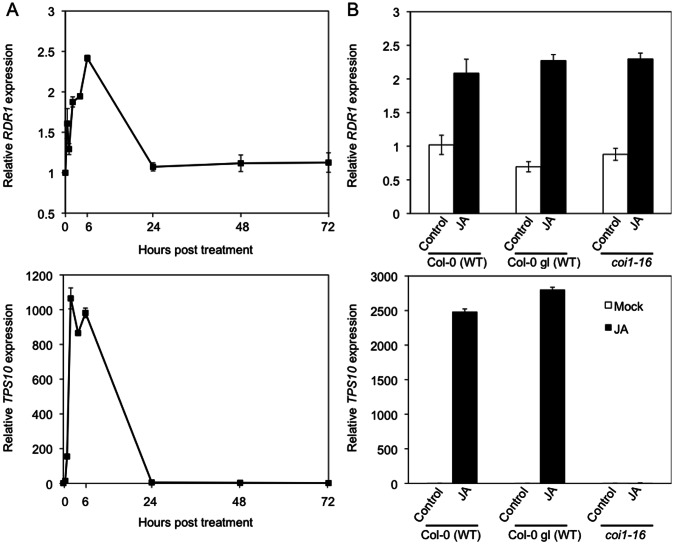
JA-induced *AtRDR1* expression is COI1-independent. (A) RTqPCR analysis of transcript accumulation for *AtRDR1* and the JA-responsive, COI1-dependent gene *AtTPS10* over 72 h following treatment of *Arabidopsis* (ecotype Col-0) plants with methyl-JA. (B) *AtRDR1* and *AtTPS10* transcript accumulation in methyl-JA (JA) or control-treated *coi1-16* mutant plants and wild-type Col-0 or Col-0 gl (the *coi1-16* background) at 6 h post-treatment. Error bars represent standard error of the mean.

JA-mediated signaling co-ordinates a large proportion of wound-induced gene expression [45]. Indeed, expression of the wound-inducible *TPS10* transcript is stimulated by treatment with methyl-JA [46]. In *N. attenuata*, *NaRDR1* expression was shown to be induced by JA [30], suggesting that the abrasion-induced expression of *AtRDR1* is regulated by JA-dependent signaling. To examine this further, wild type Col-0 plants were treated with 250 µM methyl-JA and RNAs were extracted at various times over a 72 h time-course for analysis of gene expression by Q-RT-PCT (Figure 5A). Methyl-JA treatment induced transient increases in expression of both *AtRDR1* and *TPS10*, used here as a positive control for JA-induced gene expression (Figure 5A). *AtRDR1* expression peaked at 6 h post-treatment, and by 24 h had decreased to pre-treatment levels (Figure 5A). The results show that regulation of *RDR1* gene expression by JA is conserved between *N. attenuata* and *Arabidopsis*.

To investigate further the relationship of JA-mediated signal transduction to *AtRDR1* expression, plants of the mutant line *coronatine insensitive 1–16* (*coi1-16*), which is compromised in perception of the active form of JA, JA-Ile, were treated with methyl-JA and samples were harvested at 6 h post-treatment for RNA extraction and analysis of expression of *AtRDR1* and *TPS10* by RTqPCR (Figure 5B).

It has been shown previously that the induction by methyl-JA of increased *TPS10* expression is COI1-dependent [45] and our data were consistent with this. Thus, we found that induction of *TPS10* transcript accumulation by methyl-JA was inhibited in *coi1-16* mutant plants (Figure 5B). In contrast, *AtRDR1* expression following methyl-JA treatment was similar in wild-type and *coi1-16* mutant plants (Figure 5B), demonstrating that JA-induced *AtRDR1* expression is COI1-independent.

### Ethylene and ABA Induce *RDR1* Expression

**Figure 6:**
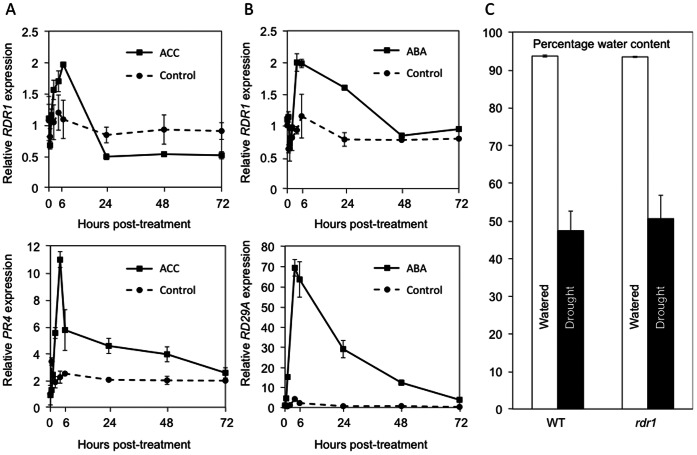
Ethylene and ABA induce *AtRDR1* expression but RDR1 is not required for drought resistance. RTqPCR analysis of expression of *AtRDR1* and the ethylene-regulated gene *AtPR4* expression in *Arabidopsis thaliana* Col-0 plants sprayed with the ethylene precursor 1-aminocyclopropane-1-carboxylic acid (ACC) at 1 mM or water (Control) (A), or of *AtRDR1* and the ABA-inducible gene *RD29A* after treatment with ABA (B) over time courses of 72 h. (C) Analysis of water content in wild-type (WT) or *rdr1*-mutant *Arabidopsis* plants watered normally (Watered) or deprived of water for 9 days (Drought). In the experiment shown, 40 well-watered plants were divided into two groups of 20, one group watered, the other subjected to drought. There was no significant difference (t-test: *p* = 0.693) in water content of water-deprived WT and *rdr1*-transgenic plants. Error bars represent standard error of the mean.

There is significant cross-talk between JA- and SA-regulated signaling and signaling mediated by ethylene and ABA [47]. Therefore, we investigated if these other stress-related phytohormones affected *RDR1* expression. *Arabidopsis* plants were treated with solutions of the ethylene precursor 1-aminocyclopropane-1-carboxylic acid (ACC) or ABA. ACC treatment induced the expression of *AtRDR1* in parallel with induction of the ethylene-responsive *AtPR4* gene peaking at 6 h post treatment (Figure 6A). ABA treatment induced *AtRDR1* expression and expression of the ABA-responsive gene *RD29A*, also with a peak in expression at 6 h post-treatment (Figure 6B).

ABA is an important regulator of drought responses [48]. As *AtRDR1* is induced by ABA treatment, this suggested the possibility that RDR1 might play a role in resistance to drought stress. However, when plants were subjected to drought by 9 days of water deprivation, there was no significant difference between the percentage water content of wild-type or *rdr1* mutant plants (Figure 6C). The same result was seen in three independent experiments.

## Discussion

Although often viewed as being SA-inducible, *RDR1* expression displays some intriguing differences, as well as similarities too, to the behaviour of other SA-regulated plant genes. As is the case with the well-studied *PR* genes, SA-induced *AtRDR1* expression was shown to be NPR1-dependent, which was confirmed using two independent *npr1* mutant lines from different *Arabidopsis* ecotypes. However, unlike the transcript for the PR1 protein, the increase in *AtRDR1* transcript accumulation was transient and its peak expression was markedly lower than that for *AtPR1*. This suggests that *AtRDR1* transcript accumulation is under tighter transcriptional and post-transcriptional control than *AtPR1*. Interestingly, SA-induced accumulation of both *AtPR1* and *AtRDR1* transcripts was diminished in plants of the SA biosynthetic mutant line *sid2* (which is compromised in *ICS1*). This led to our finding that the *ICS1* gene but not the *ICS2* gene is under a form of positive feedback from SA, the ultimate end product of ICS1 activity. The results also confirmed the primacy of ICS1 over ICS2 in facilitating SA biosynthesis in *Arabidopsis* [44].

The induction by SA of resistance to viruses is not dependent upon NPR1 [35], [36]. The dependence on NPR1 of SA-induced *AtRDR1* expression provides additional evidence, along with previous studies with transgenic plants [15], [27], that the major contribution of RDR1 to virus resistance lies in its role in basal defense, and that it is not essential for SA-induced resistance to viruses. The role of RDR1 in stress tolerance and defense via the silencing of endogenous genes is something that has been suggested previously by Pandey and Baldwin (2007) as an explanation for the susceptibility observed in *rdr1* mutant *N. attenuata* lines to herbivory [30]. Furthermore, tobacco lines deficient in RDR1 have been shown to have altered expression of other defense related genes, suggested by the authors that RDR1 plays a role in regulating other endogenous defense-related genes by suppressing the expression of regulatory molecules [33].

The JA-mediated and SA-mediated defensive signaling pathways are to a great extent antagonistic, and few transcripts are positively regulated by both [49]. Thus, the responsiveness of *RDR1* to both of these phytohormones seen in *N. attenuata* [30] and in *Arabidopsis* (this study) sets this ‘SA-responsive’ gene apart from typical SA-responsive genes like *PR1*. *RDR1* is also not a typical JA-responsive gene, since its induction by methyl-JA was not dependent upon COI1, a F-box protein responsible for degradation of JASMONATE ZIM-domain proteins that negatively regulate most JA-responsive genes [50], [51], [52]. Although most JA-responsive genes are dependent upon COI1 for induction, several, including genes involved in plant defense, have been discovered to be COI1-independent [53]. Perhaps the independence from COI1-mediated jasmonate perception allows RDR1 regulation to be outside the typical SA-JA antagonism, and may allow the gene to be similarly responsive to such a wide range of distinct stress signals as JA, SA, ethylene and ABA.

In *N. attenuata*, simulating herbivory by wounding leaves and applying oral secretions from leaf-chewing larvae caused *NaRDR1* expression to increase, due to the JA-responsiveness of the gene [30]. We found that gentle wounding, specifically the abrasion used during mechanical inoculation with virus, is sufficient to induce *AtRDR1* transcript accumulation and it was wounding, rather than an effect of the virus, that caused *AtRDR1* induction in directly-inoculated leaves. The finding is reminiscent of findings of induction by abrasion of host RDR enzyme activity in plant tissues in early studies of plant viral RNA synthesis [54]. In previous work it was suggested that induction of *RDR1* expression in inoculated and systemically infected tissues of virus-infected *Arabidopsis* was due to effects of the virus [15], [16]. However, our results indicate that these findings should be re-assessed and that *RDR1* induction in inoculated tissue was most likely due to wounding, while induction in systemically infected leaves is probably attributable to localised induction of RNA silencing, such as that which occurs during ‘green-island’ formation [55], rather than as a direct effect of the virus or its gene products.

The responsiveness of *AtRDR1* expression to a wide range of stress related hormones might imply a role in co-ordination of resistance to both biotic and abiotic insult. The responsiveness to ABA suggested that one of the stresses that RDR1 may help protect against is drought. However, *rdr1*-mutant plants were neither more nor less resistant to water loss than wild-type plants. In one way this was a surprising result because in a number of studies it has been shown that small RNA pathways affect drought responses. For example, where RNA silencing pathways have been compromised through mutation of AGO1 or DCLs 1–4, the mutant plants showed increased resistance to water loss [56], [57], [58]. Hence, although RDR1 is a component of the silencing pathway it does not appear to play a critical role in drought resistance, unlike the DCLs and AGO1. Thus, the biological implications of the ABA-responsiveness of the *RDR1* gene remain to be discovered.

## Materials and Methods

### *Arabidopsis* Mutants and Growth Conditions

*Arabidopsis thaliana* wild type Col-0, Col-0gl, and Nössen (NO) ecotypes were used in this study, either alone or alongside mutant lines with the corresponding ecotype. *Arabidopsis* mutants used included the previously characterised *NahG, sid2, npr1-1, npr1-5, coi1-16* and *rdr1, ICS1:GUS*, *ICS2:GUS*, and *35S:GUS* lines. All plants were grown in short-day condition growth chambers (Conviron Ltd., Winnipeg, Manitoba, Canada): 8 h of light at 200 micromol (photons).m^−2^.s^−1^ and 22°C, with 60% humidity.

### Hormone Treatments

Hormone treatments were conducted on plants four weeks of age. The plant hormone treatments were SA (1 mM), methyl-JA (250 µM), ABA (50 µM) and ACC (the precursor of ethylene; 1 mM) dissolved in water. Hormone concentrations were selected on the basis of previous optimization for SA and methyl-JA [46], ACC [59], and ABA [58], [60]. Control treatment used water only. Plants were sprayed until surface run-off and samples (aerial tissues of six plants) were taken and frozen in liquid nitrogen at time points of 0.5, 1, 2, 4, 6, 24, 48 and, in some experiments, 72 h post treatment. All experiments were carried out at least three times.

### Wounding Treatment

Four-week-old, wild-type Col-0 plants were wounded by squeezing leaves twice with a pair of tweezers. Two leaves per plant were wounded. Aerial plant tissue (from six plants per time point) was harvested and immediately frozen in liquid nitrogen at subsequent time points of 0.5, 1, 2, 4, 6, 24, 48 and 72 h.

### TMV Inoculation

TMV strain U1 (20 µg.ml^−1^ purified virions in sterile water) was mechanically inoculated onto Carborundum-dusted leaves. Mock-inoculation used sterile water only. *Arabidopsis* plants were inoculated at the 4- to 6-true-leaf stage. Successful inoculation was confirmed by RT-PCR on extracted plant RNA, using primers for the TMV coat protein gene (forward primer 5′-TTCTTGTCATCAGCGTGGGCCG-3′; reverse primer 5′-GCAGGACCAGAGGTCCAGACCAA-3′.

### Reverse Transcription-coupled Quantitative Polymerase Chain Reaction

Total RNA for RTqPCR analysis was extracted using TRIzol reagent (Invitrogen, Carlsbad, CA, USA) according to the manufacturer’s instructions. Total RNA was then further purified by a phenol-chloroform extraction and subsequently treated with TURBO-DNase (Ambion, Austin, TX, USA) according to the manufacturer’s instructions. First strand cDNA synthesis was carried out on 0.5 µg total RNA using GoScript (Promega, Madison, WI, USA) with random hexamer primers according to the manufacturer’s instructions. The cDNA produced was diluted 1 to 5 and RTqPCR performed using SYBR Green JumpStart Taq ReadyMix (Sigma-Aldrich, St Louis, MO, USA) in 15 µl reactions according to the manufacturer’s instructions. Reactions were conducted in triplicate. Primers sequences are given in Table 1. The gene *glyceraldehyde-3-phosphate dehydrogenase* was used as the reference gene as its expression was identified as being stable under the experimental conditions. The instrument used was a BioRad C1000 thermal cycler connected to a CFX96 Real-Time PCR Detection System and a PC running on CFX manager software (BioRad). The data was analysed using LinRegPCR [61] to give Ct and amplification efficiency values. Relative gene expression was calculated using efficiency adjusted ΔΔCt methodology, incorporating the reference transcript to control for variation in loading. Gene expression was expressed relative to mock-treated wild-type plants.

**Table 1:** Primers used for quantitative PCR.

Transcript	Locus	Forward primer 5′-3′	Reverse primer 5′-3′
*AtGAPDH*	AT1G13440	AGGCTGGGATTGCATTGAGCGA	ACACACAAACTCTCGCCGGTGT
*AtRDR1*	AT1G14790	AAGAGCGGTTCGGGCGTTGA	AGCCGAAGCCTTTGCTGACTCA
*AtPR1*	AT2G14610	CGAAAGCTCAAGATAGCCCA	AAGGCCCACCAGAGTGTATG
*AtTPS10*	AT2G24210	CTGGTGGATGGAGACAGGTT	TGAGGCTCTTGGATTTGTCC
*AtRD29A*	AT5G52310	ACCGATTCATCATCCTCTGTCCGAA	ACGTTATCGGGGTCTCGACGTT
*AtPR4*	AT3G04720	TGTTCTCCGACCAACAACTG	TGGAGCAATAAGCACTCACG
*AtICS1*	AT1G74710	CTTTTCAGTCCCTCAGGTTG	AGTTCATCATCCCAAGCAAT
*AtICS2*	AT1G18870	TGCAGTGTGAAGGACAAGAC	GAAGAGTCTCTCAGGCGTGT

*AtGAPDH* was used as the ‘housekeeping’ reference transcript. All primers (used at a final concentration of 10 pmol.µl^−1^) were designed to have an annealing temperature of 57°C.

### Generation of ICS:GUS Transgenic Lines

Generation of *ICS1:GUS*-transgenic lines was previously described by Lewsey *et al.* [46]. PCR amplification of the *ICS2* promoter region for cloning was performed using oligonucleotides designed to incorporate 5′ *Xba*I (forward primer, target sequence underlined) and 3′ *Xma*I (reverse primer, target sequence underlined) restriction sites (forward primer 5′-ATATCTAGATTAATTGTTACGAGACG-3′ and reverse primer 5′-TATCCCGGGTAGAGAGACTACGAAG-3′). The 1.5-kb product was cloned into pGEM-T Easy (Promega), sequenced to check for mutations and sub-cloned into pGreen-GUS [62] using *Xba*I and *Xma*I. The resulting plasmid was introduced into *Agrobacterium tumefaciens* GV3101::pMP90::pSOUP by electroporation and used to transform *A. thaliana* Col-0 by floral dipping [63].

### Detection of GUS Activity

Plants of four-week-old *ICS1:GUS* and *ICS2:GUS* lines were sprayed with 1 mM SA until surface run-off. As a control plants of the same age were treated with water. Aerial plant tissue was harvested at time points of 24 and 48 h post treatment for both control and SA-treated plants. Immediately after harvesting, plant tissue was stained with 5-bromo-4-chloro-3-indolyl-β-D-glucuronic acid (X-Gluc) by submerging the rosettes in 5 ml of X-Gluc solution and infiltrating them under a vacuum for 15 min. Samples were incubated at 37°C overnight. The indigo stain develops and indicates regions where the GUS reporter gene has been expressed. Samples were then soaked in 70% ethanol to remove chlorophyll.

### Drought Experiments

Water content analysis was performed according to the methods of Xu *et al.* [64]. At least 20 four-week-old plants were drenched in water for 30 min to achieve 100% soil saturation. The plants were divided into equal numbers, half receiving watering as a control, whilst the other half did not receive any more water. The position of individual plants was randomized and plants were observed daily. After 9 days without water, the aerial tissues of well-watered and drought-stressed plants were harvested and fresh weights recorded. Samples were then dried over a period of 5 days at 50°C. Dry weight was recorded and the weight loss for each plant, which is equal to water weight, was calculated. Percentage water content of each rosette was calculated by dividing the fresh weight by the water weight for each sample. This experiment was done three times.
